# Tensorial blind source separation for improved analysis of multi-omic data

**DOI:** 10.1186/s13059-018-1455-8

**Published:** 2018-06-08

**Authors:** Andrew E. Teschendorff, Han Jing, Dirk S. Paul, Joni Virta, Klaus Nordhausen

**Affiliations:** 1 CAS-MPG Partner Institute for Computational Biology, CAS Key Lab of Computational Biology, Shanghai Institute for Biological Sciences, Chinese Academy of Sciences, 320 Yue Yang Road, Shanghai, 200031 China; 2 Department of Women’s Cancer, UCL Elizabeth Garrett Anderson Institute for Women’s Health, University College London, 74 Huntley Street, London, WC1E 6BT UK; 3 UCL Cancer Institute, University College London, 72 Huntley Street, London, WC1E 6BT UK; 4 University of Chinese Academy of Sciences, 19A Yuquan Road, Beijing, 100049 China; 5 Cardiovascular Epidemiology Unit, Department of Public Health and Primary Care, University of Cambridge, Strangeways Research Laboratory, Cambridge, CB1 8RN UK; 6 University of Turku, Turku, 20014 Finland; 7 Vienna University of Technology, Wiedner Hauptstr. 7, Vienna, A-1040 Austria

**Keywords:** Multi-omic, Tensor, Dimensional reduction, Independent component analysis, mQTL, Epigenome-wide association study, Cancer

## Abstract

There is an increased need for integrative analyses of multi-omic data. We present and benchmark a novel tensorial independent component analysis (tICA) algorithm against current state-of-the-art methods. We find that tICA outperforms competing methods in identifying biological sources of data variation at a reduced computational cost. On epigenetic data, tICA can identify methylation quantitative trait loci at high sensitivity. In the cancer context, tICA identifies gene modules whose expression variation across tumours is driven by copy-number or DNA methylation changes, but whose deregulation relative to normal tissue is independent of such alterations, a result we validate by direct analysis of individual data types.

**Electronic supplementary material:**

The online version of this article (10.1186/s13059-018-1455-8) contains supplementary material, which is available to authorized users.

## Background

Omic data is now most often generated in a multi-dimensional context. For instance, for the same individual and tissue type, one may measure different data modalities (e.g. genotype, mutations, DNA methylation or gene expression), which may help pinpoint disease-driver genes [1]. Alternatively, for the same individual, the same data type may be measured across different tissues or cell types [2, 3], which may help identify the most relevant cell types or tissues for understanding disease aetiology. We refer to all of these types of multi-dimensional data generally as multi-way or multi-omic data, and when samples and molecular features are matched, the data can be brought into the form of a multi-dimensional array, formally known as a tensor [4].

While several statistical algorithms for the analysis of multi-way or tensorial data are available [4–7], their application to real data has been challenging. There are mainly three reasons for this. First, the associated multi-way datasets are often very large and how well the algorithms perform on such large sets is currently still unclear. Second, the algorithms can be computationally demanding, compromising their benefit-to-cost ratio [4]. Third, interpreting the output of these algorithms requires an in-depth understanding of the underlying methods. Exacerbating this problem, most available software packages are not user-friendly, requiring the user to have such an in-depth understanding to extract the relevant biological information. Beyond these technical challenges, there is also a lack of comparative studies, making it difficult to choose the appropriate algorithm for the task in question.

To help address some of these outstanding challenges, we here consider and evaluate a novel data tensor decomposition algorithm [8, 9], which is based on blind source separation (BSS), and specifically independent component analysis (ICA) [10]. Although common BSS techniques such as non-negative matrix factorisation and ICA have been successfully applied to a wide range of single omic data types, including e.g. gene expression [11–16], DNA methylation [17] and mutational data [18], their application to multi-way data is largely unexplored [19]. For single-omic datasets, the improved performance of ICA over non-BSS techniques like principal component analysis (PCA) is due primarily to the non-Gaussian and often sparse nature of biological sources of variation, which means that statistical deconvolution of biological samples benefits from non-linear decorrelation measures such as statistical independence (as used in ICA) [13]. It is, therefore, natural to consider analogous ICA algorithms for multi-way data, as we do here, since these may also lead to improved inference.

To assess this, we here benchmark our novel tensorial BSS algorithm against some of the most popular and powerful algorithms for inferring sources of variation from multi-omic data, including JIVE (joint and individual variation explained) [5], PARAFAC (parallel factor analysis) [4, 6], iCluster [7] and canonical correlation analysis (CCA) [20–22]. Each of these algorithms has particular strengths and weaknesses, which render comparisons between them highly non-trivial. For instance, a limitation of CCA is that it can infer only common sources of variation between data types or tissues, in contrast to JIVE or PARAFAC, which can infer both joint as well as individual sources of variation. On the other hand, JIVE and CCA can be run on multiple data matrices with different numbers of molecular features, while PARAFAC and iCluster require matched sets of features (and samples) for each data type. Model complexity also differs substantially between methods, with PARAFAC exhibiting a much lower model complexity than an algorithm such as iCluster. Thus, a comparison of all of these methods is of paramount interest, and here we do so in a tensorial context, i.e. one where the multi-way data is defined over a matched set of molecular features (e.g. genes or CpGs) and samples across all data types, allowing the data to be brought into the form of a tensor. Specifically, we shall here consider order-3 data tensors, i.e. data which can be brought into the form of an array with three dimensions (often called modes). In our evaluation and comparison of all multi-way algorithms, we consider both simulated data as well as data from real epigenome-wide association studies (EWAS). We further illustrate potential uses of our tensorial BSS algorithm (i) to detect cell-type-independent and cell-type-specific methylation quantitative trait loci (mQTLs) in multi-cell-type or multi-tissue EWAS and (ii) to detect cancer gene modules deregulated by copy-number and DNA methylation changes.

## Results

### Tensorial ICA outperforms JIVE, PARAFAC, iCluster and CCA on simulated data

**Fig. 1:**
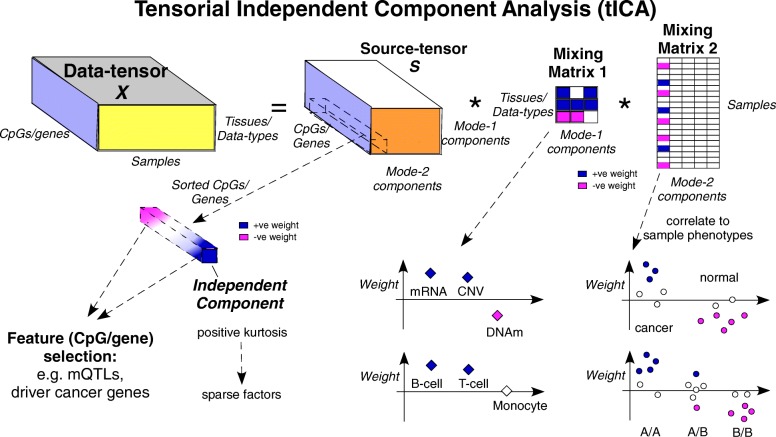
Decomposing data tensors using independent component analysis. Tensorial ICA (tICA) works by decomposing a data tensor, here depicted as an order-3 tensor with three dimensions representing features (CpGs/genes), samples and tissue or data type, into a source tensor *S* and two mixing matrices defined over tissue/data type and samples, respectively. The key property of tICA is that the independent components in *S* are as statistically independent from each other as possible. Statistical independence is a stronger criterion than linear decorrelation and allows improved inference of sparse sources of data variation. Positive kurtosis can be used to rank independent components to select the most sparse factors. The largest absolute weights within each independent component can be used for feature selection, while the corresponding component in the mixing matrices informs about the pattern of variation of this component across tissue/data types and samples, respectively. In the latter case, the weights can be correlated to sample phenotypes, such as normal/cancer status or genotype. For the first mixing matrix, the weights inform us about the relation between data types (e.g. if the copy-number change is positively correlated with gene expression), or for a multi-cell EWAS, whether mQTLs are cell type independent or not. + ve positive, −ve negative, CNV copy-number variation, DNAm DNA methylation, EWAS epigenome-wide association study, mQTL methylation quantitative trait locus, mRNA messenger RNA

Tensorial ICA (tICA) aims to infer from a data tensor statistically independent sources of data variation, which should correspond better to underlying biological factors (‘Methods’). Indeed, since biological sources of data variation are generally non-Gaussian and often sparse, the statistical independence assumption implicit in the ICA formalism can help improve the deconvolution of complex mixtures and thus, better identify the true sources of data variation (Fig. 1). As with ordinary ICA itself, there are different ways of implementing tICA, and we here consider two different flavours: tensorial fourth-order blind identification (tFOBI) and tensorial joint approximate diagonalisation of high-order eigenmatrices (tJADE) (‘Methods’). Specifically, we consider two modified versions of these, whereby tensorial PCA is applied as a noise reduction step (also called whitening) prior to implementing tICA, resulting in two algorithms we call tWFOBI and tWJADE (‘Methods’).

**Fig. 2:**
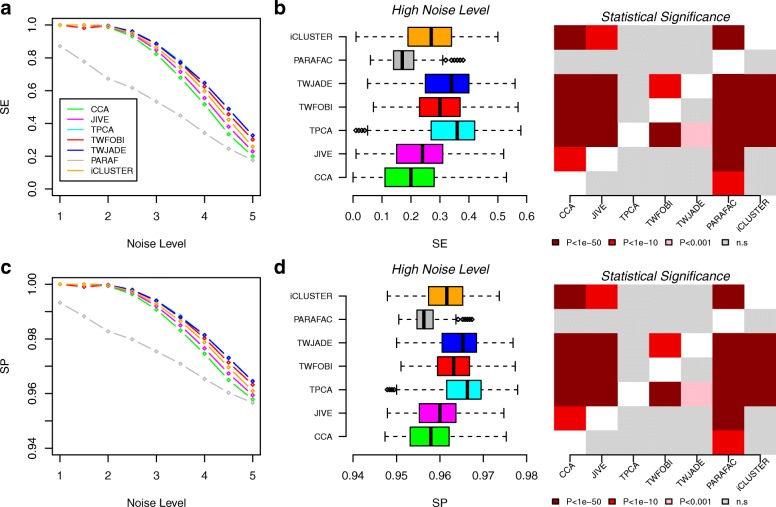
Comparison of multi-way algorithms on simulated data. **a** Sensitivity (SE) versus noise level (*x*-axis) for seven different methods as indicated, as evaluated on simulated data (data points are averages over 1000 Monte Carlo runs). In each case, the data tensor was of size 2×100×1000, i.e. two data types, 100 samples and 1000 genes. **b** Left panel: Box plots of SE values for the same seven methods for the largest noise level (5). Each box contains the SE values over the 1000 Monte Carlo runs. Right panel: Corresponding heat map of *P* values of significance for each pairwise comparison of methods. *P* values were computed from a one-tailed Wilcoxon rank sum test. For each entry specified by a given row and column, the alternative hypothesis is that the method specified in the row has a higher SE than the method specified in the column. **c,d** As **a,b**, but for the specificity (SP). SE sensitivity, SP specificity

First, we tested the two tICA algorithms, as well as tensorial PCA (tPCA), on simulated multi-way data consisting of two different data matrices defined over the same 1000 features (genes) and 100 samples (‘Methods’). The data for the two matrices was generated with a total of four sources of variation, two for each matrix, and with one source in each data matrix describing joint variation, driven by a total of 100 genes. A total of nine different noise levels were simulated, ranging from a high signal-to-noise ratio (SNR) regime (SNR = 3 and noise level = 1) to a low SNR regime (SNR = 0.6 and noise level = 5). For each noise level, a total of 1000 Monte Carlo runs were performed. In each run, we compared the multi-way algorithms in terms of their sensitivity (SE) and specificity (SP) to detect the 50 genes driving the joint variation. We did not consider the corresponding performance measures for the individual variation (i.e. the variation specific to one data type), because not all algorithms infer sources of individual variation (e.g. CCA), thus precluding direct comparison between them, and because identifying sources of joint variation is always the main purpose of multi-way algorithms. The number of components chosen for each method and the number of genes selected within components to compute SE and SP is explained in detail in ‘Methods’. SE and SP values for joint variation of each algorithm and noise level were averaged over the 1000 runs (‘Methods’). Benchmarking tICA and tPCA against PARAFAC, CCA, JIVE and iCluster, we observed that for low noise levels, all algorithms performed similarly, except PARAFAC, which exhibited significantly worse SE and SP values (Fig. 2a,c). For larger noise levels, we observed worse performance for JIVE, CCA and iCluster compared to the two different tICA methods (tWFOBI and tWJADE) (Fig. 2, ‘Methods’). Differences in SE and SP between the tICA methods and JIVE, CCA, iCluster and PARAFAC were statistically significant (Fig. 2b,d). On this data, and since tICA uses tPCA as a preliminary step, we did not observe a substantial difference between tPCA and tICA (Fig. 2). We note that in this evaluation on the simulated data, we did not consider sparse CCA (SCCA), since the sparsity itself does not optimise sensitivity and thus SCCA would perform substantially worse than CCA (data not shown). Results were unchanged if we replaced Gaussian distributions (as the sources of variation) with super-Gaussian Laplace distributions, indicating that our results are not dependent on the type of data distribution (Additional file 1: Figure S1).

### Tensorial PCA/ICA reduces running time compared to JIVE, PARAFAC and iCluster

Using the same simulated data, we further compared the algorithms in terms of their running times. A detailed comparison is cumbersome because the parameters specifying the number of components to search for are not directly comparable and differ substantially between methods. Nevertheless, using reasonable parameter choices for the simulated model above, we found that tPCA and tICA substantially speed up inference over methods such as JIVE or iCluster (Table 1). In fact, even when specifying a larger number of components for tPCA/tICA, compared to PARAFAC, JIVE or iCluster, the latter were substantially slower (Table 1), whilst also exhibiting marginally worse SE and SP values (Fig. 2). In general, we observed tICA methods to be at least 50 times faster than PARAFAC, and at least 100 times faster than JIVE and iCluster (Table 1). For much larger datasets, we found the application of iCluster to be computationally demanding and not practical. Thus, in subsequent analyses on real datasets, we decided to benchmark tPCA/tICA against PARAFAC, CCA, SCCA and JIVE.

**Table 1:** Comparison of running times of multi-way algorithms

Algorithm	Number of	Runtime (s)	Runtime (s)
	components	*n*_*g*_=1000	*n*_*g*_=2000
CCA	*K*=3	0.57±0.11	1.15±0.12
	*K*=12	0.58±0.14	1.34±0.13
JIVE	*j*_*V*_=1, *i*_*V*_=(1,1)	28.44±4.04	23.84±4.18
	*j*_*V*_=4, *i*_*V*_=(4,4)	137.77±44.94	173.20±60.35
tPCA	(2,2)	0.57±0.17	1.35±0.24
	(2,6)	0.61±0.15	1.25±0.23
tWFOBI	(2,2)	0.65±0.16	1.50±0.21
	(2,6)	0.76±0.14	1.53±0.25
tWJADE	(2,2)	0.66±0.19	1.44±0.24
	(2,6)	1.23±0.21	2.71±0.28
PARAFAC	*R*=6	22.37±2.53	37.32±4.51
	*R*=12	48.11±2.98	100.83±7.92
iCLUSTER	*K*=3	79.28±14.16	595.06±70.67
	*K*=12	114.28±30.02	688.74±166.85

Seven multi-way algorithms in terms of the running times to infer components of variation (runtime) in the simulation model considered in Fig. 2. Estimates are medians and median absolute deviations over 100 Monte Carlo runs for when the signal-to-noise ratio is 1 (i.e. noise level = 3 in Fig. 2). The second column specifies the parameter values for the number of components used in each algorithm. The first rows for each method are as follows. For CCA, three sets of canonical vector pairs (*K*=3) are shown. For JIVE, the rank of joint variation (*j*_*V*_=1) and rank of individual variation (*i*_*V*_=1) for each data type are shown. For TPCA, TWFOBI and TWJADE, we inferred two components for both the data type and sample dimensions. For PARAFAC, the rank of decomposition was *R*=6 and for iCLUSTER the maximum number of clusters *K* was set to 3. For the second rows, the total number of components is exactly matched (12) for all methods. The running times are reported for two scenarios differing in the number of genes *n*_*g*_, as indicated, and were obtained on a Dell PowerEdge R830 with Intel Xeon E5-4660 v4 2.2GHz processors

### tICA exhibits improved power in a real multi-tissue smoking EWAS

**Fig. 3:**
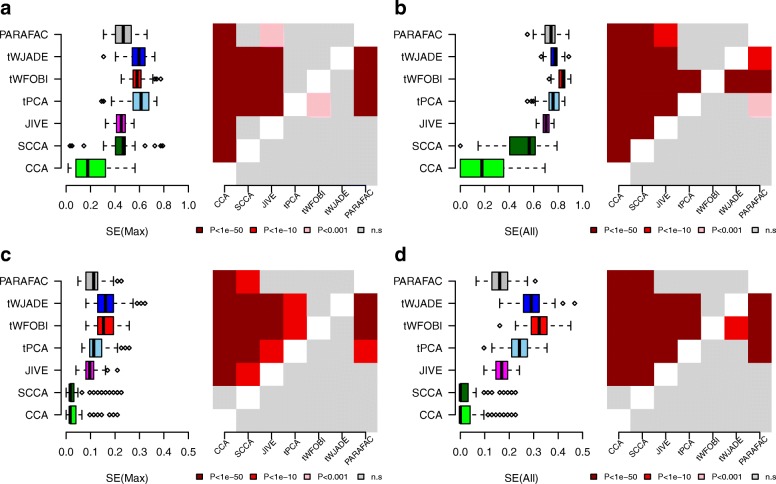
Comparison of multi-way algorithms on a multi-tissue smoking EWAS. **a** Left panel: Box plot of sensitivity (SE) values for each of the seven methods as applied to the data tensors of dimension 2×152×1062 (two tissues, 152 samples and 1000 randomly selected non-smkDMCs plus 62 smkDMCs) and for 100 different selections of non-smkDMCs. SE(Max) is the maximum sensitivity to capture 62 smkDMCs among all inferred components. Right panel: Heat map of the corresponding one-tailed paired Wilcoxon rank sum test, benchmarking the SE values of each method (*y*-axis) against each other method (*x*-axis). **b** As **a**, but now for the combined sensitivity (SE(All)) obtained from all enriched components. **c,d** As **a**,**b**, but now for data tensors of dimension 2×152×10 062 and for 100 randomly selected 10 000 non-smkDMCs. EWAS epigenome-wide association study, SE sensitivity, smkDMC smoking-associated differentially methylated CpG, SP specificity

Next, we asked if tPCA/tICA also leads to improved power on real data. An objective evaluation on real data is challenging due to the difficulty of defining a gold-standard set of true positive associations. Fortunately, however, a meta-analysis of several smoking EWAS in blood has demonstrated that smoking-associated differentially methylated CpGs (smkDMCs) are highly reproducible, defining a gold-standard set of 62 smkDMCs (‘Methods’) [23]. Recently, we also showed that effectively all 62 smkDMCs are associated with smoking exposure if DNA methylation (DNAm) is measured in buccal samples [2]. Thus, one way to compare algorithms objectively is in terms of their sensitivity to identify these 62 smkDMCs in a matched blood-buccal EWAS consisting of Illumina 450k DNAm profiles for a total of 152 women (‘Methods’, [2]). Because there are two distinct samples (one blood plus one buccal) per individual, most of the variation is genetic. Hence, to reduce this background genetic variation, we first computed the SE values on a reduced data matrix obtained by combining the 62 smkDMCs with 1000 randomly selected non-smoking associated CpGs (a total of 100 Monte Carlo randomisations). We considered both the maximum SE value attained by a component, as well as the overall SE obtained by combining selected CpGs from components significantly enriched for smkDMCs (‘Methods’). This revealed that JIVE, CCA/SCCA and PARAFAC were all superseded by tPCA and tICA (Fig. 3a,b). Differences between tPCA and tICA were generally not significant (Fig. 3a), although tWFOBI attained higher combined SE values than tPCA and tWJADE (Fig. 3b).

Next, we scaled up the data matrices by combining the 62 smkDMCs with a larger set of 10 000 non-smkDMCs, recomputing the SEs (again for 100 different Monte Carlo selections of 10 000 non-smkDMCs). As expected, with an increase in the number of CpGs, the SE of all algorithms dropped, likely driven by increased confounding due to genetic variation (Fig. 3c,d). With the increase in probe number, tICA (tWFOBI and tWJADE) outperformed not only JIVE, PARAFAC and CCA/SCCA, but also tPCA (Fig. 3c,d), in line with the increased sparsity of the smoking-associated source of variation.

**Fig. 4:**
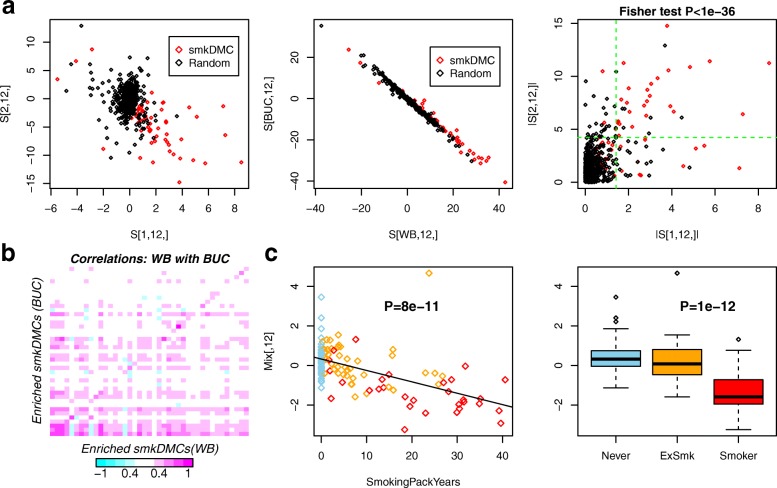
Validation of tensorial ICA on multi-tissue smoking EWAS. **a** Left panel: Scatterplot of the weights of estimated independent components *S*_1,12,*i*_ and *S*_2,12,*i*_ from the data tensor of dimension 2×152×1062, with mode 1 representing tissue type, mode 2 the different women and mode 3 the CpGs. Red denotes the smkDMCs. Middle panel: As left panel, but now for the rotated tensor, projecting the data onto the whole blood (WB) and buccal (BUC) dimensions, demonstrating the strong correlation between the DNAm variation in whole blood and buccal tissue. Right panel: As left panel, but now for the absolute weights. The green dashed lines represent the cutoff point selecting the 62 CpGs with the largest absolute weights. There are in total 41 smkDMCs among the three larger quadrants, corresponding to a sensitivity of 41/62=0.66, with the enrichment *P* value given above the plot. **b** Pearson correlation heat map of the 41 smkDMCs between whole blood (WB) and buccal (BUC) tissue, with correlations computed over the 152 samples. **c** Plots of the 12th independent component of the mixing matrix in the sample space (*y*-axis) against smoking exposure for the 152 samples. Left panel: Smoking-pack-years. Right panel: Smoking status (never smokers, ex-smokers and smokers at sample draw). *P* values are from linear regressions. BUC buccal, DNAm DNA methylation, EWAS epigenome-wide association study, ExSmk ex-smoker, smkDMC smoking-associated differentially methylated CpG, WB whole blood

To illustrate how the output produced by tICA can be used for valuable inference, we focus on a particular Monte Carlo run and a specific component (estimated using tWJADE), which obtained a high sensitivity for smkDMCs (component 12, Fig. 4a). We note that the two independent components (ICs) *S*_1,12,*i*_ and *S*_2,12,*i*_ exhibited a less correlative structure than the corresponding components projected onto the blood and buccal dimensions, demonstrating that tWJADE does indeed identify components that are less statistically dependent (Fig. 4a). Confirming the high sensitivity of these ICs, the 62 smkDMCs were highly enriched among CpGs with the largest absolute weights in any one of the two ICs (Fig. 4a, Fisher test *P*<1×10^−36^ and SE=41/62∼0.66). We further verified that the 41 enriched smkDMCs exhibited strong Pearson correlations between their DNAm profiles in blood and buccal, as required since smoking exposure is associated with similar DNAm patterns in these two tissue types (Fig. 4b) [2]. Further confirming that component 12 is associated with smoking exposure, we correlated the weights of the corresponding column of the estimated mixing matrix with two different measures of smoking exposure, demonstrating in both cases a strong association (Fig. 4c). Thus, application of tICA on DNAm data results in components that are readily interpretable in terms of their associations with known smoking exposure across features and samples.

### tICA identifies mQTLs in a multi-cell-type EWAS

**Fig. 5:**
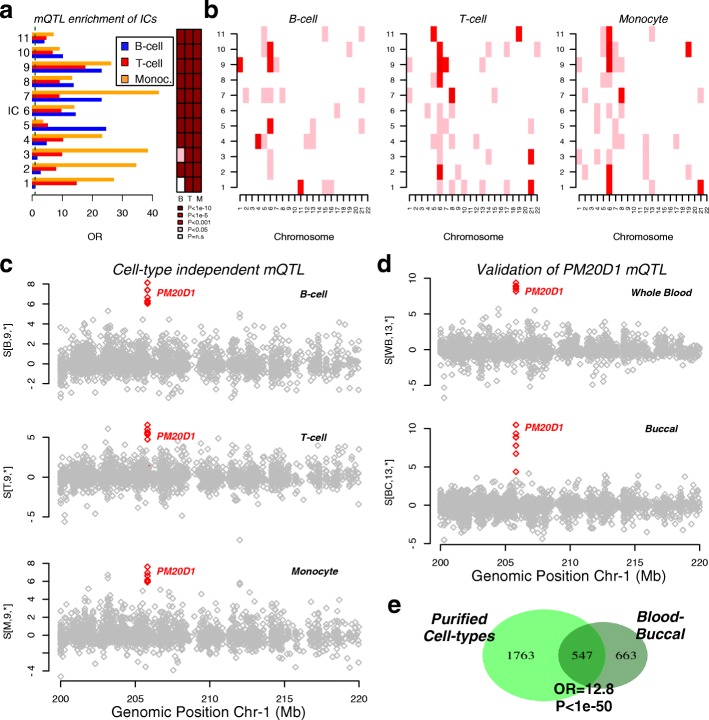
Tensorial ICA identifies components enriched for mQTLs in an EWAS of purified cell types. **a** Left panel: Bar plot of the odds ratio (OR) of enrichment of the top-ranked 500 CpGs for mQTLs in each of the 11 ICs and cell types, as indicated. Right panel: Corresponding heat map indicating the *P* values of enrichment as estimated using a one-tailed Fisher’s exact test. **b** Heat maps of enrichment *P* values of the top-ranked 500 CpGs from each IC for chromosomes. The significance of *P* values is indicated in different colours using same scheme as in **a**. **c** An example of a cell-type-independent mQTL mapping to chromosome 1. Plots show the weights of the corresponding components for B cells, T cells and monocytes, with the selected CpGs mapping to the mQTL indicated in red. **d** Validation of the mQTL in **c** in an independent blood-buccal EWAS. **f** Venn diagram showing the overlap of mQTLs derived from the ICs in the purified cell-type EWAS with those derived from the blood-buccal EWAS. The odds ratio (OR) and one-tailed Fisher test *P* value of the overlap are given. Chr chromosome, EWAS epigenome-wide association study, IC independent component, mQTL methylation quantitative trait locus, OR odds ratio

Having established the better performance of tICA over other state-of-the-art methods, we next considered the application of tICA (specifically tWFOBI) in an EWAS of 47 healthy individuals, for which three purified cell types (B cells, T cells and monocytes) have been profiled with Illumina 450k DNAm bead arrays [3] (‘Methods’). We chose tWFOBI over tWJADE because of its computational efficiency (Table 1). Given that three cell types were measured for each individual, the expectation is that a significant amount of inter-individual variation in DNAm would correlate with genetic variants (i.e. mQTLs) [24]. Thus, it is important to evaluate the ability of tICA to detect mQTLs and to determine whether these are blood-cell-subtype specific or not. Applying tWFOBI to the data tensor for the 3 cell types × 47 samples × 388 618 probes, we inferred a total of 11 ICs in the sample-mode space (yielding 33 ICs across sample and cell-type modes combined). For each of these 11 ICs in each cell type, we ranked probes according to their absolute weights and tested the enrichment of the top-500 probes against a high-quality list of 22 245 mQTLs as derived in [25] (‘Methods’). This high-confidence list of mQTLs all passed a very stringent unadjusted *P* value threshold of *P*=1×10^−14^ in each of five different human cohorts, encompassing five different age groups [25]. We observed strong statistical enrichment for mQTLs in many ICs (Fig. 5a). We also tested separately for enrichment of chromosomes. This revealed enrichment, notably of chromosomes 6 and 21, but also of 1, 4, 7 and 8 (Fig. 5b). For instance, IC-9 was enriched for mQTLs and chromosome 1 in all three cell types (Fig. 5a,b). Supporting this, we found a clear example of a cell-type-independent mQTL mapping to the 1q32 locus of the *PM20D1* gene (Fig. 5c), a major genome-wide association study (GWAS) locus associated with Parkinson’s disease [26]. Focusing on chromosome 6, another cell-type-independent mQTL mapped to *MDGA1* (Additional file 1: Figure S2), a major susceptibility locus for schizophrenia [27]. Other mQTLs driving ICs were cell type specific, e.g. mQTLs mapping to *ATXN1* and *SYNJ2* were dominant in the ICs projected along B cells, but not among T cells or monocytes (Additional file 1: Figure S3). Although assessing whether mQTLs are truly cell type independent or cell type specific is not possible without genotype information, we nevertheless estimated, based on the IC weight distribution of the mQTLs across cell types, that approximately 75% of the mQTLs enriched in ICs were cell type independent (Additional file 1: Figure S4). This estimate of the non-specificity of blood-cell-subtype mQTLs is similar to that obtained by a previous study (≥79*%*) using neutrophils, monocytes and T cells [28].

Next, we validated the mQTLs found using an independent dataset. Thus, we applied tWFOBI to the blood-buccal EWAS considered earlier. We inferred a source tensor of dimension 2×26×447 259, i.e. a total of 52 ICs, defined over two tissue types and 26 components in sample-mode space. As before, we observed very strong enrichment, notably for the same chromosomes 6 and 21 (Additional file 1: Figure S5). The previously found mQTL at the *PM20D1* locus was also prominent in one of the inferred ICs in this blood-buccal EWAS, confirming its validity and further supporting that this mQTL is cell type independent (Fig. 5d). Overall, from the pure blood-cell-subtype EWAS, we detected a total of 1763 mQTLs, of which 547 were also observed in the blood-buccal EWAS (odd ratio = 12.8, Fisher test *P*<1×10^−50^, Fig. 5e). Thus, we can conclude that tWFOBI is able to identify components of variation across cell types and samples that capture a significant number of mQTLs, without matched genotype information.

### tICA outperforms JIVE and PARAFAC in their sensitivity to detect mQTLs

**Fig. 6:**
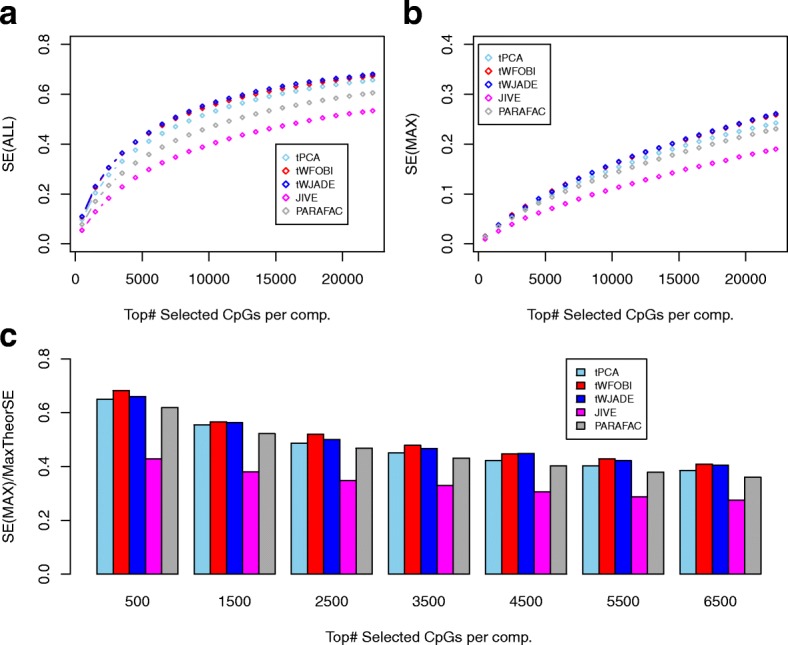
tICA outperforms JIVE and PARAFAC in detecting mQTLs. **a** Plot of the overall sensitivity (SE(ALL), *y*-axis) against the number of top-ranked CpGs selected in a component (*x*-axis) for five different algorithms. **b** As **a**, but now for the maximum sensitivity attained by any single component (SE(MAX), *y*-axis). **c** Bar plot of the maximum sensitivity attained by any single component expressed as a fraction of the maximum possible value given the number of selected top-ranked CpGs per component. mQTL methylation quantitative trait locus, SE sensitivity

Given the ability of tICA to detect mQTLs, we next benchmarked the performance of all algorithms in terms of their sensitivity to detect mQTLs in the EWAS of the three purified blood cell subtypes considered earlier. Because of the presence of three cell types, for this analysis we excluded CCA and sCCA since these methods are designed for only two data matrices. As before, we computed two sensitivity measures to detect the 22 245 mQTLs from the Aries database [25], designed to assess the overall sensitivity across all inferred components, and another designed to assess the maximum sensitivity attained by any single component. Varying the number of top-ranked selected CpGs in components from 500 up to 22 245, we observed that over the whole range, tFOBI and tJADE were optimal, clearly outperforming both PARAFAC and JIVE (Fig. 6a). The maximum sensitivity attained by any individual component was also best for the tICA methods (Fig. 6b). To better evaluate the enrichment of these components for mQTLs, we also considered the ratio of the sensitivity to the maximum possible sensitivity, recording the maximum value attained by any component. This demonstrated that when selecting the top-500 CpGs, the components inferred using tICA could capture over 60% of the maximum possible number of mQTLs, i.e. over 60% of the 500 CpGs mapped to mQTLs (Fig. 6c). In contrast, JIVE components contained only just over 40% of mQTLs (Fig. 6c). We note that although the performance of JIVE could be significantly improved by also including the components of individual variation, that approximately 80% of mQTLs have been estimated to be independent of blood cell subtype [28], supporting the view that JIVE is less sensitive in capturing cell-type-independent mQTLs. All these results were stable to repeated runs of the algorithms, as only PARAFAC exhibited variation between runs. However, this variation was relatively small (Additional file 1: Figure S6).

Next, we repeated the same sensitivity analysis to detect mQTLs in our buccal-blood EWAS, now also including CCA and sCCA (as there are only two tissue/cell types). Confirming the previous analysis, tICA methods outperformed JIVE and PARAFAC by over 20% in terms of the overall sensitivity, whilst also attaining a better sensitivity at the individual component level (Additional file 1: Figure S7). Of note, the sensitivity of both CCA and sCCA was substantially worse, since mainly only the top canonical vector was significant.

### Application of tICA to multi-omic cancer data reveals dosage-independent effects of differentially expressed genes

**Fig. 7:**
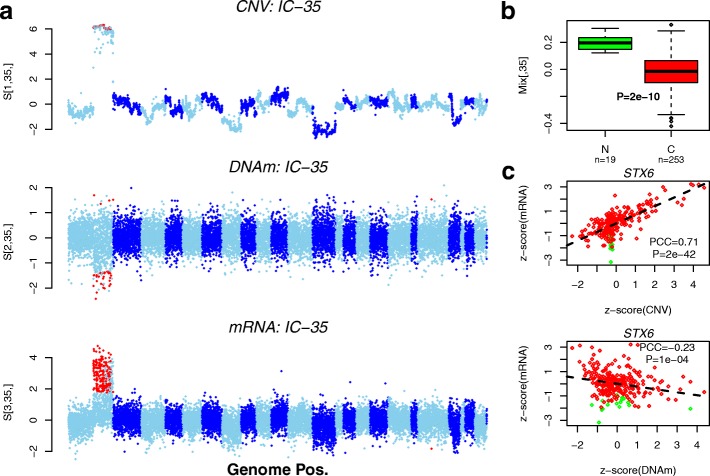
Validation of tICA on a multi-omic cancer set. **a** Manhattan-like plots of IC-35 in gene space, as inferred using tWFOBI on the colon TCGA set, projected along the CNV, DNAm and mRNA axes. Red points highlight genes that had large weights in both CNV and mRNA dimensions (CNV), in both DNAm and mRNA dimensions (DNAm), and the union of these (mRNA). Chromosomes are arranged in increasing order and displayed in alternating colours. **b** Box plots of the corresponding weights of IC-35 in the sample space, discriminating normal colon (N) from colon cancer (C). *P* value is from a Wilcoxon rank sum test. **c** Scatterplots of a driver gene (*STX6*) between *z*-score normalised segment level (CNV) and mRNA expression (top panel) and between *z*-score normalised DNAm level and mRNA expression (lower panel). Colours indicate normal (green) and cancer (red). The regression line, Pearson correlation coefficient and *P* value are shown. C cancerous, CNV copy-number variation, DNAm DNA methylation, IC independent component, mRNA messenger RNA, N normal, PCC Pearson correlation coefficient, Pos. position

To demonstrate further the ability of tICA to retrieve interesting patterns of variation in a multi-omic context, we applied it to the colon cancer The Cancer Genome Atlas (TCGA) dataset [1], comprising a matched subset of copy-number variation (CNV), DNAm and RNA-seq data over 13 971 genes and 272 samples (19 normals plus 253 cancers) [29]. We applied tWFOBI to the resulting 3×272×13 971 data tensor, inferring a total of 3×37 ICs, which were ranked in order of decreasing kurtosis (‘Methods’). Of the 37 ICs, 20 correlated with normal/cancer status (*P*<0.05/37∼0.001), with four of these capturing correlations between CNV and gene expression (Additional file 1: Table S1). All four ICs were strongly enriched for specific chromosomal bands (Additional file 1: Table S1), in line with those reported in the literature [1, 30], and one of these (IC-35) also exhibited concomitant correlation between DNAm and gene expression (Additional file 1: Table S1). Plotting the weights of IC-35 along the CNV, DNAm and mRNA axes confirmed the ability of tWFOBI to identify patterns of mRNA expression variation, which are driven by local CNV and which also associate with local variation in DNAm (Fig. 7a). The corresponding weights along the sample mode confirmed the association with normal/cancer status (Fig. 7b). Scatterplots of the *z*-score normalised CNV and DNAm patterns against gene expression for one of the main driver genes (*STX6*) confirmed the strong associations between CNV/DNAm and mRNA expression (Fig. 7c). Strikingly, we observed that while variations in copy number and DNAm of *STX6* modulate expression differences between colon cancers, that the deregulation of *STX6* expression between normal and cancer is clearly independent of copy-number and DNAm state (Fig. 7c).

**Fig. 8:**
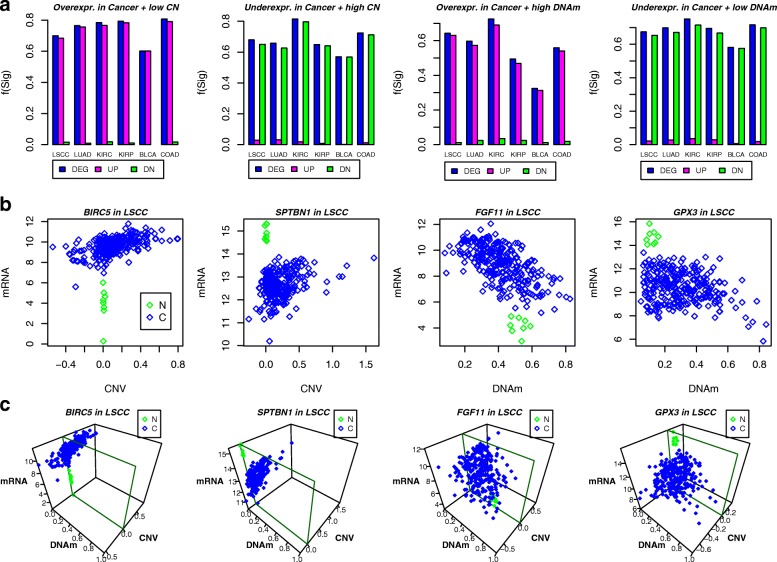
Multi-dimensional patterns of differential expression in cancer. **a** Box plots of the fraction of differentially expressed genes in cancer, which remain differentially expressed when specific cancer subsets are compared to normal-adjacent samples, for six different TCGA cancer types (LSCC, LUAD, KIRC, KIRP, BLCA and COAD), and for four different scenarios: genes overexpressed in cancer and considering cancers with neutral or copy-number loss of that gene (first panel), genes underexpressed in cancer and considering cancers with neutral or copy-number gain (second panel), genes overexpressed in cancer and considering cancers with the highest levels of gene promoter DNAm (third panel), and finally genes underexpressed in cancer and considering cancers with the lowest levels of gene promoter DNAm (fourth panel). In each panel, blue denotes the fraction of over/underexpressed genes that are differentially expressed when only the specific cancer subset is compared to the normal samples. Magenta denotes the fraction that are overexpressed and green denotes the fraction that are underexpressed. **b** Scatterplots of mRNA expression against either copy-number variation level (CNV) or DNAm level for selected genes in LSCC. The selected genes represent examples of genes from **a**. For instance, BIRC5 in LSCC is overexpressed in cancer compared to normal, and this overexpression relative to normal is independent of the CNV of the cancer. **c** As **b**, but the 3D scatterplots also display the CNV or DNAm level. These plots illustrate that the difference in expression between cancer and normal is also independent of the other variable (e.g. CNV or DNAm). For instance, the underexpression of GPX3 in LSCC is neither driven by promoter DNAm nor by CNV losses. BLCA bladder adenocarcinoma, C cancerous, CN copy number, CNV copy-number variation, DEG differentially expressed gene, COAD colon adenocarcinoma, DNAm DNA methylation, KIRC kidney renal cell carcinoma, KIRP kidney papillary carcinoma, LSCC lung squamous cell carcinoma, LUAD lung adenocarcinoma, mRNA messenger RNA, N normal, Overexpr. overexpression, Underexr. underexpression

To validate this important finding and determine the extent of this phenomenon, we analysed five additional TCGA datasets (see ‘Methods’), but now using a more direct approach. For each TCGA set, we first identified the subset of differentially expressed genes (DEGs) between normal and cancer (adjusted *P* value threshold of 0.05) that also exhibit a positive correlation between expression and copy number as assessed over cancers only, i.e. we selected those DEGs with a CNV-dosage effect across cancers. For those overexpressed in cancer, we then asked if individual tumours exhibiting a neutral CNV state (the CNV state of the normal samples) or a CNV loss still exhibited overexpression relative to the normal samples. Remarkably, we observed that a very high fraction of these DEGs remained overexpressed when restricting to the subset of cancer samples with low or neutral CNV, thus indicating that *their overexpression in cancer is not dependent on CNV state*, despite their expression across individual cancer samples being modulated by CNV state (Fig. 8a). This pattern of differential expression being independent of CNV state was also seen for DEGs with a CNV-dosage effect across tumours and which were underexpressed in cancer. Indeed, restricting to cancers with neutral or copy-number gain (Fig. 8a), these genes were generally still underexpressed in these cancer samples compared to normal tissue. Similar patterns were observed when DEGs were selected for DNAm-expression dosage effects across tumours (Fig. 8a). Specific examples for lung squamous cell carcinoma (LSCC) confirmed that DEGs in LSCC that exhibit a CNV or DNAm dosage effect across tumours exhibit differential expression that is not dependent on CNV or DNAm state (Fig. 8b,c). Thus, these data support the finding obtained using tICA, demonstrating the value and power of tICA to extract biologically important and novel patterns of data variation in a multi-omic context.

## Discussion

Here we have assessed and benchmarked a novel suite of tensorial decomposition algorithms (tPCA, tWFOBI, and tWJADE) against a number of state-of-the-art alternatives. Specifically, while popular multi-way algorithms such as JIVE, iCluster or CCA/SCCA are in principle applicable to non-tensorial multi-way data (e.g. if the features across data types are distinct or not matched), when assessed in a tensorial context (i.e. when all dimensions are matched), these established methods are outperformed by the tensorial PCA and ICA methods considered here. This was demonstrated not only on simulated data, but also in the context of two real EWAS, where tICA methods were significantly more powerful in detecting differentially methylated CpGs associated with an epidemiological factor (smoking) and single-nucleotide polymorphisms (SNPs; mQTLs). For a real EWAS, tICA also outperformed tPCA, in line with the fact that biological sources of data variation are non-Gaussian and sparse, and therefore, more readily identified using statistical independence as a (non-linear) deconvolution criterion (as opposed to the linear decorrelation criterion used in tPCA). Thus, this extends the improvements seen for ICA over PCA on ordinary omic data matrices [13, 16] to the tensorial context. In addition, tPCA and tICA offer substantial (50–100-fold) speed advantages over methods like iCluster, JIVE and PARAFAC, which can become computationally demanding or even prohibitive. Further application of tICA to a multi-cell-type EWAS (B cells, T cells and monocytes) revealed its ability to identify loci enriched for cis-mQTLs (as cis-mQTLs make up over 90% of validated mQTLs in the Aries database [25]). Indeed, tICA achieved relatively high sensitivity values with top-ranked CpGs in components containing over 60% mQTLs. Given that here we were limited because we did not have access to matched genotype information, our results demonstrate the potential of tICA to detect mQTLs in the absence of such genotype information. For instance, it identified many cell-type-independent mQTLs, of which a substantial proportion have been validated in an independent blood-buccal EWAS study, and with several mapping to key GWAS loci for important diseases like Parkinson’s and schizophrenia. Although most of the identified mQTLs were blood cell type independent, tICA estimated that approximately 25% of mQTLs may be blood cell type specific, in line with the estimate of 20% obtained by Blueprint using a slightly different combination of blood cell subtypes (neutrophils, monocytes and T cells) [28]. We note that application of tICA to any multi-cell-type or multi-tissue EWAS is likely to have components strongly enriched for mQTLs, since for the same individuals, DNAm is being measured in at least two different tissues or cell types, and therefore, genetic effects that do not depend on cell type are bound to explain most of the inter-individual variation [24, 31]. Thus, we conclude that tICA could be an extremely versatile tool for identifying novel candidate mQTLs in multi-cell EWAS for which matched genotype information may not be available. tICA may also help to identify groups of widely separated mQTLs that are regulated by the same SNP and bound e.g. by a common transcription factor [32].

More generally, tICA can be applied to any multi-way data tensor to identify complex patterns of variation correlating with phenotypes of interest and the underlying features driving these variation patterns. This is accomplished by first correlating inferred ICs of variation in the sample-mode space with sample phenotype information (e.g. age, smoking, normal/cancer status and genotype) and subsequently selecting the features with the largest weights in these correlated components. As an illustrative example, the application of tICA to a multi-omic TCGA dataset revealed a deep novel insight: namely, that most DEGs in cancer *that exhibit a CNV or DNAm dosage-dependent effect on expression across individual tumours* exhibit differential expression relative to the normal tissue in a manner that does not, in fact, depend on CNV or promoter DNAm state. In other words, although CNV and DNAm variation strongly modulates expression variation of these DEGs across individuals tumours, for most of the genes exhibiting this CNV or DNAm dosage-dependent expression pattern, their deregulation relative to normal cells appears to be independent of the underlying CNV or promoter DNAm state. Although it is clear that differential expression in cancer can be the result of many mechanisms other than CNV or DNAm, our observation is significant, because we did not just select cancer DEGs, but the subset of these that exhibit a CNV or DNAm dosage-dependent effect on expression across tumours. The implications of our observation are important, given that many cancer classifications have been derived from unsupervised (clustering) analyses that were performed using only tumours, thus ignoring their patterns of variation relative to the normal reference state. Other large cancer studies, such as METABRIC [33], which did not profile normal tissue samples, identified novel candidate oncogenes and tumour suppressors solely based on CNV-dosage effects on gene expression across cancers, yet our results indicate that this could identify many false positives in the sense that their overexpression or underexpression in cancer is not dependent on the underlying CNV state. We point out that although this finding could have been obtained without application of a multi-way algorithm, that this would have required substantial prior insight. Therefore, this subtle pattern of variation across multiple data types was only discovered thanks to applying an agnostic method like tICA.

Although we have shown the value of tICA in identifying mQTLs and interesting patterns of variation across different data types in cancer-genome data, it is also important to discuss some of the limitations, which, however, also apply to all the other multi-way algorithms considered here. In particular, identifying sources of DNAm variation associated with epidemiological factors in a multi-tissue EWAS setting can be difficult due to confounding genetic variation. Indeed, in our application to a buccal-blood Illumina 450k EWAS, we found that the sensitivity of all algorithms dropped very significantly if they were applied to all ∼480 000 CpGs. Thus, it is important to devise improvements to these tensorial methods. For instance, one solution may be to first perform dimensional reduction using supervised feature selection on separate data types, and subsequently applying the tensorial methods on a reduced feature space. Alternatively, supervised tensorial methods, such as tensorial slice inverse regression [34], may help to identify sources of variation specifically associated with epidemiological variables.

## Conclusion

In summary, the combined tPCA and tICA methods presented here will be an extremely valuable tool for analysis and interpretation of complex multi-way data, including multi-omic cancer data, as well as for the detection and clustering of mQTLs in multi-cell-type EWAS where genotype information may not be available.

## Methods

Below we briefly describe the main tensorial BSS algorithms [8, 9, 35] as implemented here. For more technical details, see [8, 9, 35]. We also provide brief details of our implementation of JIVE, PARAFAC, iCluster, CCA and SCCA. All these implementations are available as R functions within Additional file 2.

### Tensorial PCA

We assume that we have *i*=1,…,*p* independent and identically distributed realisations of a matrix $X_{i}\in \mathbf {R}^{p_{1}\times p_{2}}$, which can be structured as an order-3 data tensor *X* of dimension *p*_1_×*p*_2_×*p*. Then, tPCA decomposes *X* as follows:

1 $$ X=S\odot_{m=1}^{2}\Omega_{m},  $$

where *S* is also a 3-tensor of dimension *p*_1_×*p*_2_×*p* and *Ω*_*m*_ (*m*=1,2) are orthogonal *p*_*m*_×*p*_*m*_ matrices, i.e. $\Omega _{m}^{T}\Omega _{m}=I_{p_{m}}$. Here, ⊙ denotes the tensor contraction operator. For instance, for *Z* an *r*-tensor of dimension *p*_1_×⋯×*p*_*r*_ and *A* a matrix of dimension *p*_*m*_×*p*_*m*_, *Z*⊙_*m*_*A* describes the *r*-tensor with entries $(Z\odot _{m}A)_{i_{1} \ldots i_{m} {\ldots } i_{r}}=Z_{i_{1} {\ldots } j_{m} {\ldots } i_{r}}A_{i_{m}j_{m}}\phantom {\dot {i}\!}$ where the Einstein summation convention is assumed (i.e. indices appearing twice are summed over, e.g. $M_{ik}M_{in}=\sum _{i}{M_{ik}M_{in}}=\left (M^{T}M)_{kn}\right)$. Thus, $S\odot _{m=1}^{2}\Omega _{m}$ is a 3-tensor with entries

2 $$ \left(S\odot_{m=1}^{2}\Omega_{m}\right)_{i_{1}i_{2}i}=S_{k_{1}k_{2}i}(\Omega_{1})_{i_{1}k_{1}}(\Omega_{2})_{i_{2}k_{2}}.  $$

In the above tPCA decomposition, the entries $S_{k_{1}k_{2}}$ are assumed to be linearly uncorrelated. Introducing the operator ⊙_−*m*_, which for general *r* is defined in entry form by

3 $$ (X\odot_{-m}X)_{uv}=X_{i_{1} {\ldots} i_{m-1}ui_{m+1} {\ldots} i_{r}i}X_{i_{1} {\ldots} i_{m-1}vi_{m+1} {\ldots} i_{r}i}  $$

uncorrelated components, means that the covariance matrix *S*⊙_−*m*_*S*=*Λ*_*m*_ is diagonal of dimension *p*_*m*_×*p*_*m*_. Its entries are the ranked eigenvalues of the *m*-mode covariance matrix (*X*⊙_−*m*_*X*), which can be expressed as

4 $$ (X\odot_{-m}X)=\Omega_{m}\Lambda_{m}\Omega_{m}^{T}.  $$

These ranked eigenvalues are useful for performing dimensional reduction, i.e. projecting the data onto subspaces carrying significant variation. For instance, one could use random matrix theory (RMT) [17, 36] on each of the *m*-mode covariance matrices above to estimate the appropriate dimensionalities *d*_1_,…,*d*_*r*_. This would lead to a tPCA decomposition of the form $X=S\odot _{m=1}^{2}\Omega ^{(R)}_{m}$, with *S* a *d*_1_×*d*_2_×*p* tensor and each $\Omega ^{(R)}_{m}$ a reduced matrix obtained from *Ω*_*m*_ by selecting the first *d*_*m*_ columns. We note that for any of the original dimensions *p*_1_,…,*p*_*r*_ that are small, such dimensional reduction is not necessary.

In the applications considered here, our data tensor *X* is typically of dimension *n*_*t*_×*n*_*s*_×*n*_*G*_, where *n*_*t*_ denotes the number of data or tissue types, *n*_*s*_ the number of samples and *n*_*G*_ the number of features (e.g. genes or CpGs). We note that the tPCA decomposition is performed on the first two dimensions (typically data type and samples), so there are two relevant covariance matrices. In the special case of a data matrix (a 2-tensor), standard PCA involves the diagonalisation of one data covariance matrix.Hence, for a 3-tensor, there are two data covariance matrices, and for an (*r*+1)-tensor, there are *r*. Here we use tPCA as implemented in the tensorBSS R package [37].

### tICA: the tWFOBI and tWJADE algorithms

For a data tensor $X\in \mathbf {R}^{p_{1}\times \dots \times p_{r}\times p}$, the tICA model is

5 $$ X = S\odot_{m=1}^{r}\Omega_{m},  $$

but now with the *p*_1_,…,*p*_*r*_ random variables $S_{k_{1} {\ldots } k_{r}}\in \mathbf {R}^{p}$ ($S\in \mathbf {R}^{p_{1}\times {\ldots } \times p_{r}\times p}\phantom {\dot {i}\!}$) mutually statistically independent and satisfying $\operatorname {E}[\!S_{k_{1} {\ldots } k_{r}}]=0$ and $\operatorname {Var}[\!S_{k_{1} {\ldots } k_{r}}]=I$. We note that *X* could be a suitably dimensionally reduced version *X*^(*R*)^ of *X*, such as that obtained using tPCA. For instance, in our applications, *X*^(*R*)^ would typically be a 3-tensor of dimension *n*_*t*_×*d*_*S*_×*n*_*G*_ where *d*_*S*_<*n*_*S*_. This dimensional reduction, and optionally the scaling of variances, is known as whitening (W).

As with ordinary ICA, there are different algorithms for inferring mutually statistical ICs $S_{k_{1} {\ldots } k_{r}}$. One algorithm is based on the concept of simultaneously maximising the fourth-order moments (kurtosis) of the ICs (since by the central limit theorem, linear mixtures of these are more Gaussian and therefore, have smaller kurtosis values). This approach is known as fourth-order blind identification (FOBI) [38]. Alternatively, one may attempt a joint approximate diagonalisation of higher order eigenmatrices (JADE) [35, 39]. We note that although we use the tFOBI and tJADE functions in tensorBSS, that these do not implement tPCA beforehand. Hence, in this work we implement modified versions of tFOBI and tJADE, which include a prior whitening transformation with tPCA. We call these modified versions tWFOBI and tWJADE.

### Benchmarking of tPCA and tICA against other tensor decomposition algorithms

JIVE (joint and individual variation explained) [5] is a powerful decomposition algorithm that identifies both joint and individual sources of data variation, i.e. sources of variation that are common and specific to each data type. For two data types (i.e. two tissue types or two types of molecular features), three key parameters need to be specified or estimated to run JIVE. These are the number of components of joint variation (*dJ*) and the number of components of variation that are specific to each data type (*dI*_1_ and *dI*_2_). On simulated data, these parameters are chosen to be equal to the true (known) values, i.e. for our simulation model, *dJ*=1, *dI*_1_=1 and *dI*_2_=1. In our real-data applications, *dJ* is estimated using RMT on the concatenated matrix obtaining by merging the two data type matrices together (after *z*-score normalising features to make them comparable), whilst *dI*_*i*_ are estimated using RMT [17]. We note that these are likely upper bounds on the true number of individual sources of variation that are not also joint. We implemented JIVE using the r.jive R package available from http://www.r-project.org.

PARAFAC (parallel factor analysis) [4, 6] is a tensor decomposition algorithm in which a data tensor is decomposed into the sum of *R* terms. Each term is a factorised outer product of rank-1 tensors (i.e. vectors) over each mode. Thus, the one key parameter is *R*, which is the number of terms or components in the decomposition. In our simulation model, we chose *R*=4. Although one of the two sources of variation in each data type is common to both (hence, there are three independent sources), we nevertheless ran PARAFAC with one additional component to assess its ability to infer components of joint variation more fairly. In the real-data applications, we estimated *R* as $\sum _{i=1}^{n_{t}}{dI_{i}}-dJ$ (with *n*_*t*_ the number of tissue or cell types), since this should approximately equal the total number of independent sources of variation. We implemented PARAFAC using the multiway R package available from http://www.r-project.org.

iCluster [7] is a joint clustering algorithm for multi-way data. It models joint and individual sources of variation as latent Gaussian factors. The key parameter is *K*, which is the total number of clusters to infer. Although for the simulated data there were only three independent sources of variation, we chose *K*=4 to assess more fairly the ability of the algorithm to infer the joint variation (choosing *K*=3 would force the algorithm to find the source of joint variation). We implemented iCluster using the iCluster R package available from http://www.r-project.org. CCA [20] and its sparse version, sparse CCA (sCCA/SCCA) [21, 22], are methods to identify joint sources of variation (called canonical vectors) between two data matrices, where at least one of the dimensions is matched across data types. Here we implement the version of CCA and sCCA in the R package PMA available from http://www.r-project.org. One key parameter is *K*, the maximum number of canonical vectors to search for. Another parameter is the number of permutations used to estimate the significance of the covariance of each of the *K* canonical vectors. In each permutation, one of the data matrices is randomised (say by permuting the features around) and CCA/sCCA is reapplied. Since the data matrices are typically large, the distribution of covariances for the permuted cases is very tight. Thus, even 25 permutations are sufficient to estimate the number of significant canonical vectors reasonably well. The number of significant canonical vectors was defined as the number of components that exhibit observed covariances larger than the maximum value obtained over all 25 permutations, and is, thus, bounded above by *K*. In the non-sparse case, the two penalty parameters were chosen to be equal to 1, which means no penalty term is used. For sCCA, we estimated the best penalty parameters using an optimisation procedure, as described in [21, 22], with the number of permutations set to 25 and the number of iterations equal to 15. On the simulated data, we ran CCA with *K*=3, as *K* needs to specify only the maximum number of components to search for (the actual number of significant canonical vectors is one in our instance, as we have one source of joint variation). In the real-data applications, we chose *K* to be equal to *dJ*, as estimated using the procedure for JIVE, and used a larger number of iterations (50) per run.

### Evaluation on simulated data

Here we describe the simulation model. The model first generates two data matrices of dimension 1000×100, representing two data types (e.g. DNA methylation and gene expression) where rows represent features and columns samples. We assume that the column and row labels (i.e. samples and genes) of the two matrices are identical and ordered in the same way. We assume one source of individual variation (IV) for each data matrix, each driven by 50 genes and 10 samples with the 50 genes and 10 samples unique to each data matrix. We also assume one source of common variation driven by a common set of 20 samples. The genes driving this common source of variation, however, are assumed distinct for each data matrix. In total, there are 100 genes (50 for each data matrix) associated with this joint variation (JV). For the 50 genes driving the JV in one data type and the 20 samples associated with this JV, we draw the values from a Gaussian distribution $\mathcal {N}(e,\sigma)$, whereas for the other 50 genes in the other data type, we draw them from $\mathcal {N}(-e,\sigma)$, all with *e*=3 and *σ* representing the noise level. Likewise for the IV, we use Gaussians $\mathcal {N}(e,\sigma)$. The rest of the data is modelled as noise $\mathcal {N}(0,\sigma)$. We consider a range of nine noise levels, with *σ* ranging from 1 to 5 in steps of 0.5. Thus, at *σ*=3, the SNR=*e*/*σ*=1. For each noise level, we perform 1000 Monte Carlo runs, and for each run and algorithm, we estimate SE and SP for correctly identifying the 100 genes driving the JV.

For tPCA, tWJADE and tWFOBI, SE and SP were calculated as follows. We inferred a total of 12 components over the combined data type and sample modes (2 in data type mode × 6 in sample space). We then projected the inferred components onto the original data-type dimensions, using the inferred 2×2 mixing matrix. For each data type and each of the six components, we then selected the top-ranked 50 genes by absolute weight in the component. This allowed us to compute a SE and SP value for each data type and component. For each component, we then averaged the SE and SP values over the two data types. In the last step, we select the component with the largest SE and SP value and record these values. We note that the resulting SE and SP values are not dependent on choosing 12 components. As long as the number of estimated components is larger than the total number of components of variation in the data (which for the simulated data is four), the results are invariant to the number of inferred components.

For CCA, which can only infer sources of joint variation, we ran it to infer a number of components (*K*=3) larger than the true number (there is only one source of JV). Pairs of canonical vectors were then selected according to whether their joint variance is larger than expected, as assessed using permutations. From hereon, the procedure to compute SE and SP proceeds as for the other algorithms, by selecting the component with the best SE and SP value. As with the other methods, the results do not depend on how we choose *K* as long as *K* is larger than or equal to 1.

For PARAFAC, we ran it to infer *R*=4 components. Since for PARAFAC there is only one inferred projection across features per component, for each component we rank the features according to their absolute weight, select the top-ranked 50, and then compute two separate SE (or SP) values, one for each of the two sets of 50 true positive genes driving JV. We then select for each set of JV driver genes, the component achieving the best SE (or SP). Finally, we average the SE and SP values for the two sets of true positives. As with the other algorithms, the results do not depend on the choice of *R*, as long as *R* is larger then or equal to 4 (since there are four sources of variation, two of IV and two of JV, which counts as two in the PARAFAC setting). For JIVE, we ran it to infer one source of JV and two sources of IV. Because JIVE stacks the data matrices corresponding to the two data types together, we then select the 100 top-ranked genes, ranked by absolute weight in the inferred JV matrix. SE and SP are then computed. Once again, the results are stable to choosing a larger number of inferred sources of JV, because for the simulated data there is only one source of JV. Further details for all methods can be found in Additional file 2. Finally, for each algorithm and noise level, SE and SP are averaged over all the 1000 Monte Carlo runs. Finally, the statistical significance of the SE and SP values between algorithms was assessed using paired non-parametric Wilcoxon rank sum tests. The whole analysis above was repeated for sources of variation drawn from a Laplace distribution (with the same mean and standard deviation as the Gaussians above), to capture the super-Gaussian nature of real biological data better.

### Illumina 450k DNA methylation and multi-way TCGA datasets

We analysed Illumina 450k datasets from three main sources. One dataset is a multi-blood-cell subtype EWAS derived from 47 healthy individuals and three cell types (B cells, T cells and monocytes) [3]. Specifically, we used the same normalised data as used in [3], with the resulting data tensor being of dimension 3×47×388 618, after removing poor quality probes and probes with SNPs [40].

Another dataset was generated in [2]. It consists of two tissue types (whole blood and buccal), 152 women and 447 259 probes, resulting in a data tensor of dimension 2×152×447 259. After quality control, and after removing probes on the X and Y chromosomes, polymorphic CpGs, probes with SNPs at the single-base extension site and probes containing SNPs in their body as determined by Chen et al. [40], we were left with 447 259 probes.

Finally, we also analysed six datasets from TCGA. Specifically, we processed the RNA-seq, Illumina 450k DNAm and copy-number data for six different cancer types: colon adenocarcinoma (COAD), lung adenocarcinoma (LUAD), lung squamous cell carcinoma (LSCC), kidney renal cell carcinoma (KIRC), kidney papillary carcinoma (KIRP) and bladder adenocarcinoma (BLCA). All of these contained a reasonable number of normal-adjacent samples. The processing was carried out following the same procedure described by us in [29], which resulted in data tensors over three data types (mRNA, DNAm and copy number), 14 593 common genes and the following sample numbers: 273 cancers and 8 normals for LSCC, 390 cancers and 20 normals for LUAD, 292 cancers and 24 normals for KIRC, 195 cancers and 21 normals for KIRP, 194 cancers and 13 normals for BLCA, and 253 cancers and 19 normals for COAD. We note that although these numbers of normal samples are small, that these are the normal samples with data for all three data types.

### Identifying smoking-associated CpGs in the multi-tissue (whole blood + buccal) EWAS

To test the algorithms on real data, we considered the matched multi-tissue (whole blood and buccal) Illumina 450k DNAm dataset for 152 women [2]. Smoking has been shown to be reproducibly associated with DNAm changes at a number of different loci [23]. We, therefore, used as a true positive set a gold-standard list of 62 smkDMCs, which have been shown to be correlated with smoking exposure in at least three independent whole blood EWAS [23]. The 62 smkCpGs were combined with 1000 randomly selected CpGs (non-smoking-associated), resulting in a data tensor of dimension 2×152×1062. Robustness was assessed by performing 1000 different Monte Carlo runs, each run with a different random selection of 1000 non-smoking associated CpGs. The whole analysis was then repeated for 10 000 randomly selected CpGs (data tensor of dimension 2×152×10 062) and for a total of 1000 different Monte Carlo runs. For the tPCA/tICA algorithms, the dimensionality parameters were chosen based on RMT as applied on the two separate matrices. Specifically, estimated unmixing matrices were of dimension 2×2 (for tissue-type mode) and *d*×*d* (for sample mode) with *d* the maximum of the two RMT estimates obtained from each tissue-type matrix.

SE to capture the 62 smkCpGs was calculated in two different ways. In one approach, we used the maximum SE attained by any IC, denoted SE(max), whilst in the other approach, we allowed for the possibility that different enriched ICs could capture different subsets of smkCpGs. Thus, in the second approach, the SE was estimated using the union of the selected CpGs over all enriched ICs. We note that enrichment of ICs for the smkCpGs was assessed using a simple binomial test and selecting those with a *P* value less than the Bonferroni corrected value (i.e. less than 0.05 per number of ICs). In both approaches, the CpGs selected per component were the 62 with the largest absolute weights in the component, i.e. the number of selected CpGs was matched to the number of true positives.

For JIVE, the number of components of joint variation was determined by applying RMT to the data matrix obtained by concatenating the features of the blood and buccal sets together with features standardised to unit variance to ensure comparability between data types. For the number of components of individual variation, we used the RMT estimates of each individual dataset, as this provides a safe upper bound. For PARAFAC, the number of components was determined by the sum of the RMT estimates for the blood and buccal sets separately minus the value estimated for the concatenated matrix, as we reasoned that this would best approximate the total number of components of variation across the two data types (joint or individual). For CCA and sCCA, the maximum number of canonical vectors to search for was set to be equal to the RMT estimate of the concatenated matrix, i.e. equal to the dimension of joint variation used in JIVE. For all methods, we selected the top-ranked 62 CpGs with the largest absolute weights in each component, and estimated SE using the same two approaches described above for tPCA/tICA.

### mQTL and chromosome enrichment analysis

We applied tWFOBI to the data tensor of a multi-cell-type EWAS (Illumina 450k) for 47 healthy individuals and three cell types (B cells, T cells and monocytes) [3]. Specifically, we used the same normalised data as used in [3], i.e. a data tensor of dimension 3×47×388 618, after removing poor quality probes and probes with SNPs [40]. Using RMT [17], we estimated a total of 11 components in the sample-mode space, and so we inferred a source tensor of dimension 3×11 618, and mixing matrices of dimension 3×3 and 11×11. We also applied tWFOBI to the previous blood plus buccal DNAm dataset, but for all 447 259 probes that passed quality control. Applying RMT, we estimated 26 significant components in the sample space. Hence, we applied tWFOBI on the 2×152×447 259 data tensor to infer a source tensor of dimension 2×26×447 259 and mixing matrices of dimension 2×2 and 26×26. For both datasets, and for each inferred IC, we selected the 500 probes with the largest absolute weights and tested enrichment of mQTLs against a high-confidence mQTL list from [25] (22 245 mQTLs). This list was generated as the overlap of mQTLs (passing a stringent *P* value threshold of 1×10^−14^) in blood derived from five different cohorts representing five different age groups. Odds ratios and *P* values of enrichment were estimated using Fisher’s exact test. For chromosome enrichment, we obtained *P* values using a binomial test. In selecting the top-500 probes from each component, we note that this threshold is conservative, as all inferred ICs exhibited positive kurtosis with kurtosis values that remained significantly positive after removing the top-500 ranked probes.

To obtain estimates of cell-type-independent and cell-type-specific mQTLs, we used the following approach. The first mode/dimension of the estimated source tensor was rotated back to the original cell types, using the estimated mixing matrix (of dimension 3×3, since there were three cell types). For each of the previously enriched mQTLs, we compared its weights in all three components, each component being associated with a given cell type. For instance, if *S*_*t*,*cp*,∗_ denotes the component *cp* for cell type *t*, thus defining a vector of weights over all CpGs, we asked if the absolute weight of the given mQTL CpG is large for all cell types or not. If it was sufficiently large (i.e. if within the top 10% quantile of the weight distribution) for all cell types, it was declared to be cell type independent. If the mQTL weight for one or two cell types fell within the lower 50% quantile of weights, we declared it a cell-type-specific mQTL.

We also performed a comparative analysis of all multi-way algorithms in terms of their sensitivity to detect mQTLs, as given by the high-confidence list of 22 245 mQTLs from the Aries database [25]. To assess the stability of the conclusions, we computed SE as described earlier, but considered a range of top selected CpGs per component, ranging from 500 up to 22 245 in units of 500. As before, we estimated the overall SE by taking into account the union of all selected CpGs from each component, as well as the maximum SE attained by any single component. Since the SE attained by any single component is bounded by the number of selected CpGs, we also considered the SE normalised for the number of selected CpGs.

### Application of tICA to multi-omic cancer data

We used the same normalised integrated copy-number state (segment values), Illumina 450k DNAm and RNA-seq datasets of six cancer types from TCGA [1], as used in our previous work [29]. For the cancer types considered, see above. We initially applied tWFOBI to the colon adenocarcinoma TCGA dataset, estimating unmixing matrices of dimension 3×3 (for data type) and *K*×*K* (for sample mode) where *K* was the maximum RMT estimate over each of the three data-type matrices. Features driving each IC in each data-type dimension were selected using an iterative approach in which genes were ranked by absolute weight, and recursively removed until the kurtosis of the IC was less than 1, or the number of removed genes was larger than 500. Genes selected in common between the CNV and mRNA modes, or between the DNAm and mRNA modes, were declared driver genes between the respective data types. To identify components correlating with normal/cancer status, we obtained the mixing matrix of the samples and then correlated each component to normal/cancer status using Wilcoxon’s rank sum test.

## Additional files

Additional file 1 Contains all supplementary figures and supplementary tables. (DOCX 14322 kb)

Additional file 2 A file containing R scripts for the tPCA, tWFOBI, tWJADE, CCA, sCCA, PARAFAC, JIVE and iCLUSTER algorithms as implemented in this work. (R 17 kb)
