# Supplementary material for: Tensorial blind source separation for improved analysis of multi-omic data
Source: Genome Biol. 2018 Jun 8;19:76. doi: 10.1186/s13059-018-1455-8 (PMC5994057; doi:10.1186/s13059-018-1455-8)
Supplement: Supplementary file 1 — Contains all supplementary figures and supplementary tables. (DOCX 14322 kb) [file 13059_2018_1455_MOESM1_ESM.docx]

# ADDITIONAL FILE 1

# Tensorial Blind Source Separation for Improved Analysis of Multi-Omic Data

Andrew E. Teschendorff ^1,2,3^ , Jing Han^1,4^, Dirk Paul^5^, Joni Virta^6^  and Klaus Nordhausen^7^

1. CAS Key Laboratory of Computational Biology, CAS-MPG Partner Institute for Computational Biology, 320 Yue Yang Road, Shanghai 200031, China.

2. Department of Women’s Cancer, University College London, 74 Huntley Street, London WC1E 6AU, United Kingdom.

3. Statistical Cancer Genomics, Paul O’Gorman Building, UCL Cancer Institute, University College London, 72 Huntley Street, London WC1E 6BT, United Kingdom.

4. University of Chinese Academy of Sciences, 19 Yuquan Road, Beijing 100049, China.

5. Department of Public Health and Primary Care, University of Cambridge, Strangeways Research Laboratory, CB1 8RN. Cambridge, UK.

6. University of Turku, 20014. Turku, Finland.

7. Vienna University of Technology, Wiedner Hauptstr.7, A-1040, Vienna, Austria.

*Corresponding author: Andrew E. Teschendorff- [a.teschendorff@ucl.ac.uk](mailto:a.teschendorff@ucl.ac.uk) , [andrew@picb.ac.cn](mailto:andrew@picb.ac.cn)

**This documents contains all Supplementary Figures and Supplementary Tables**

**SUPPLEMENTARY FIGURES**


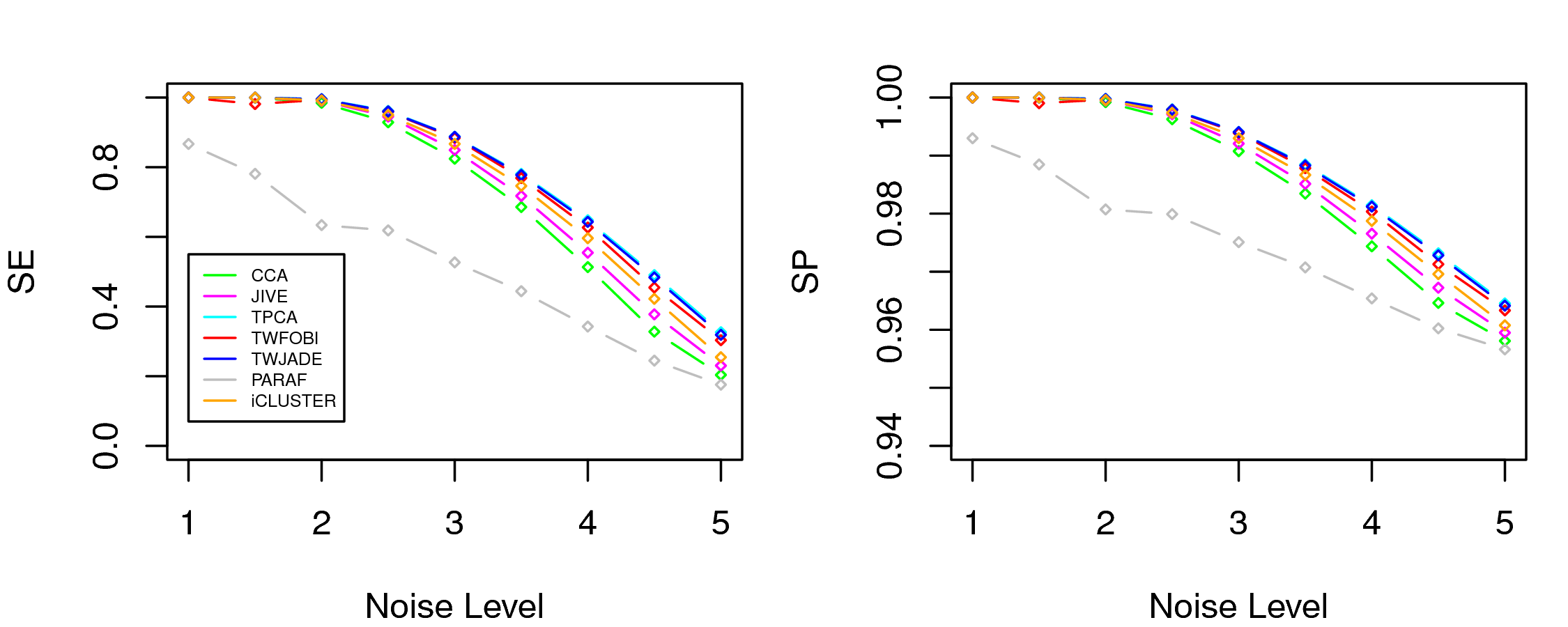
**SI fig.S1: Comparison of multi-way algorithms on simulated data (Laplacian distribution).** Sensitivity (SE) and Specificity (SP) versus noise level (x-axis) for 7 different methods as indicated, as evaluated on simulated data (data points are averages over 1000 Monte-Carlo runs). In each case the data-tensor was of size 2 x 100 x 1000. In this instance, sources of variation were generated from a Laplace distribution, to model distributions with longer tails than Gaussians.


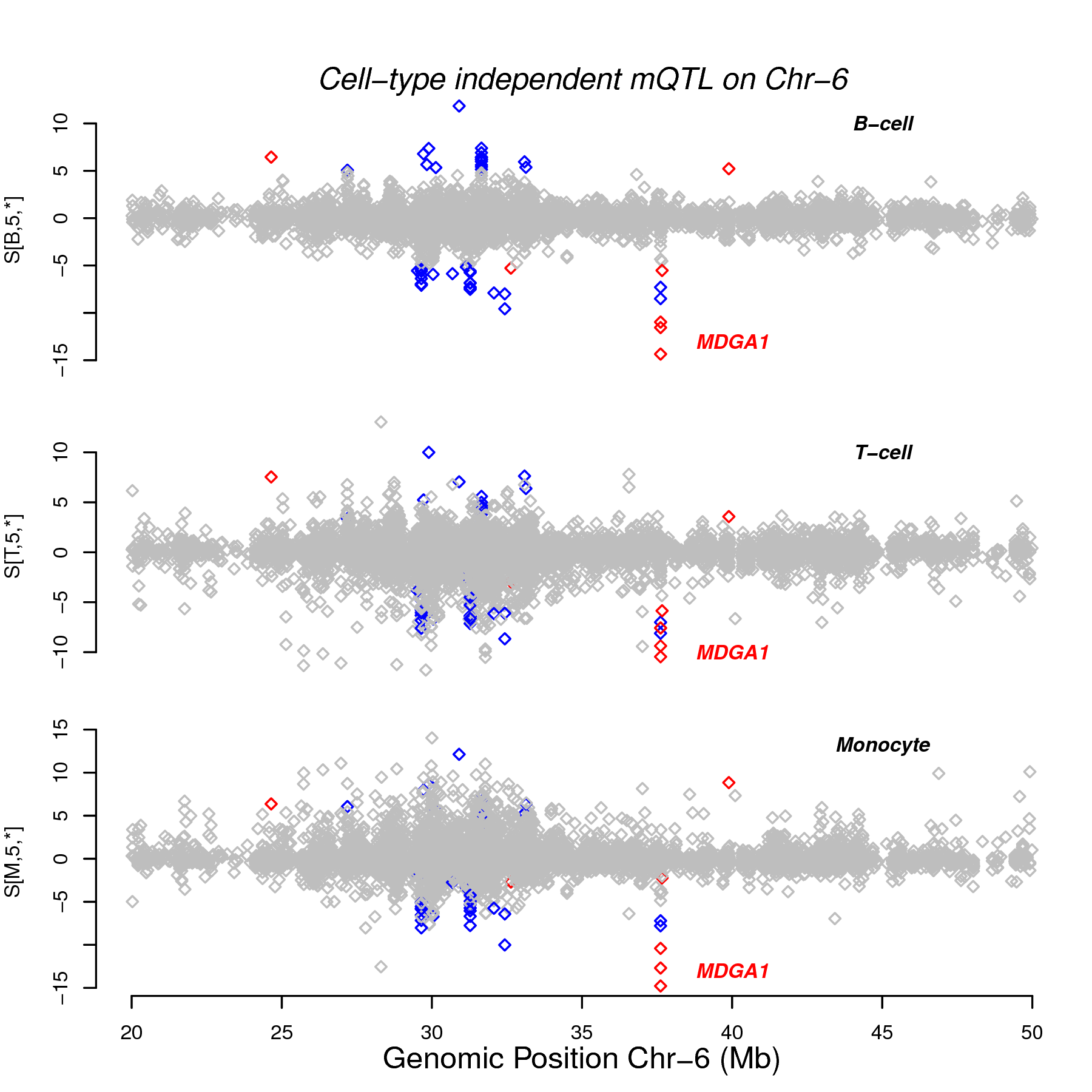
**SI fig.S2: Blood cell subtype independent mQTL inferred using tWFOBI.** Scatterplots of the weights of IC-5 along each cell-type dimension, against genomic position on chromosome-6. CpGs indicated in blue were selected in B-cells as those with largest absolute weights among the top-ranked 500. In red, we indicate those mapping to mQTLs. Observe how most of the CpGs driving IC-5 in B-cells are also driving variation in the corresponding IC along T-cells and Monocytes, and that the main driver are mQTLs mapping to the MDGA1 gene locus.


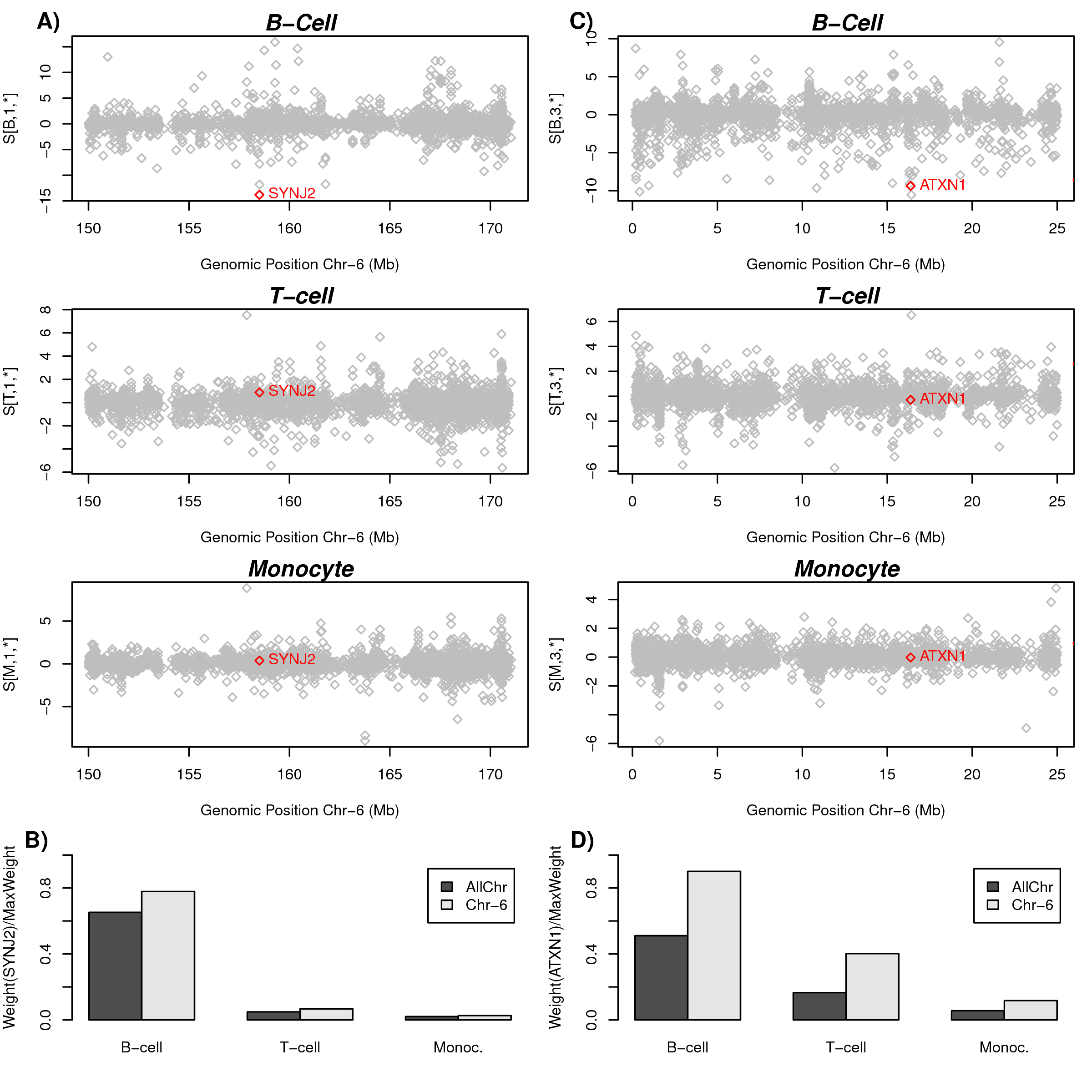
**SI fig.S3: Blood cell subtype specific mQTLs inferred using tWFOBI. A-C)** Two examples of blood cell-subtype specific mQTLs mapping to SYNJ2 and ATXN1 on chromosome-6. Both mQTLs appear to be specific to B-cells, as the corresponding weights in the independent components are relatively large only for the component projected along B-cells. **B-D)** Corresponding relative absolute weight fraction for the corresponding mQTLs as measured relative to the maximum absolute weight in the same independent component (AllChr), or relative to the maximum absolute weight in the component when restricting to CpGs on chromosome-6. Observe how the specific mQTL has a much larger relative weight fraction in B-cells compared to T-cells or Monocytes, suggesting that these mQTLs are specific to B-cells.

**
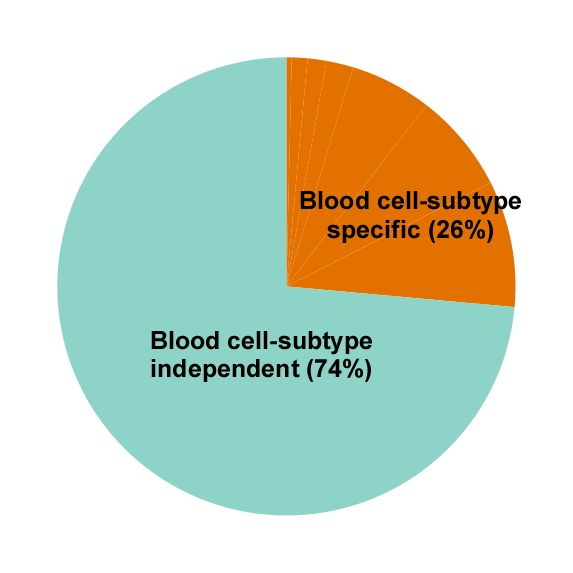
**

**SI fig.S4: Estimated fractions of blood cell-subtype independent and cell-subtype specific mQTLs.** Using the Illumina 450k EWAS dataset over 47 healthy individuals and 3 blood cell subtypes (T-cells, B-cells and Monocytes), we provide an estimate of the relative fraction of cell-type independent and cell-type specific mQTLs, where cell-subtype independent refers to mQTLs with large absolute weights in all 3 independent components aligned along the cell-type dimensions (B-cells, T-cells and Monocytes). Any mQTL with large absolute weights in an independent component associated with 1 or 2 cell-type only, is referred to as cell-subtype specific.


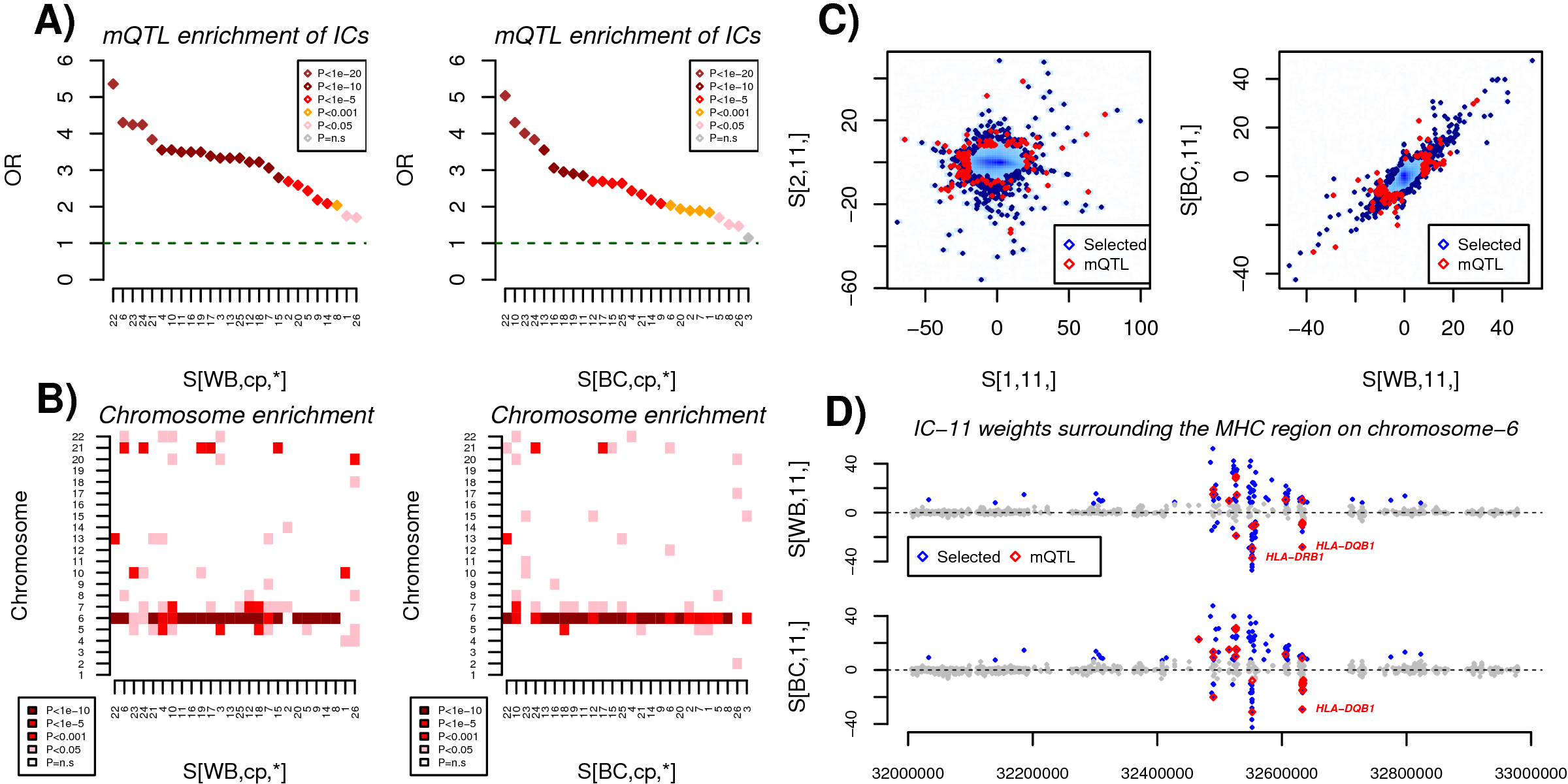
**SI fig.S5: Application of tWFOBI to blood-buccal EWAS. A)** Components inferred by tWFOBI ranked by Odds Ratio (OR) of enrichment for mQTLs, with P-values of significance estimated using a one-tailed Fisher-test, for the 2 different tissue types: whole blood (WB) (left) and buccal (BC) (right). **B)** Corresponding enrichment heatmaps of components for chromosomes, with P-values derived from a binomial test, as indicated. **C)** Left panel: scatterplots of the weights in IC-11 for the two dimensions in tissue-space., with blue dots denoting selected CpGs (those with largest weights) and red denoting selected mQTLs. Right panel: as left, but now for IC-11 aligned along the whole blood and buccal dimensions. Note how the components are uncorrelated in the left panel but correlated in the right, as required by the statistical independence criterion of tICA. **D)** Example of a region on Chr-6, enriched for selected CpGs in IC-11 and also enriched for mQTLs mapping to the major-histocompatibility complex locus.


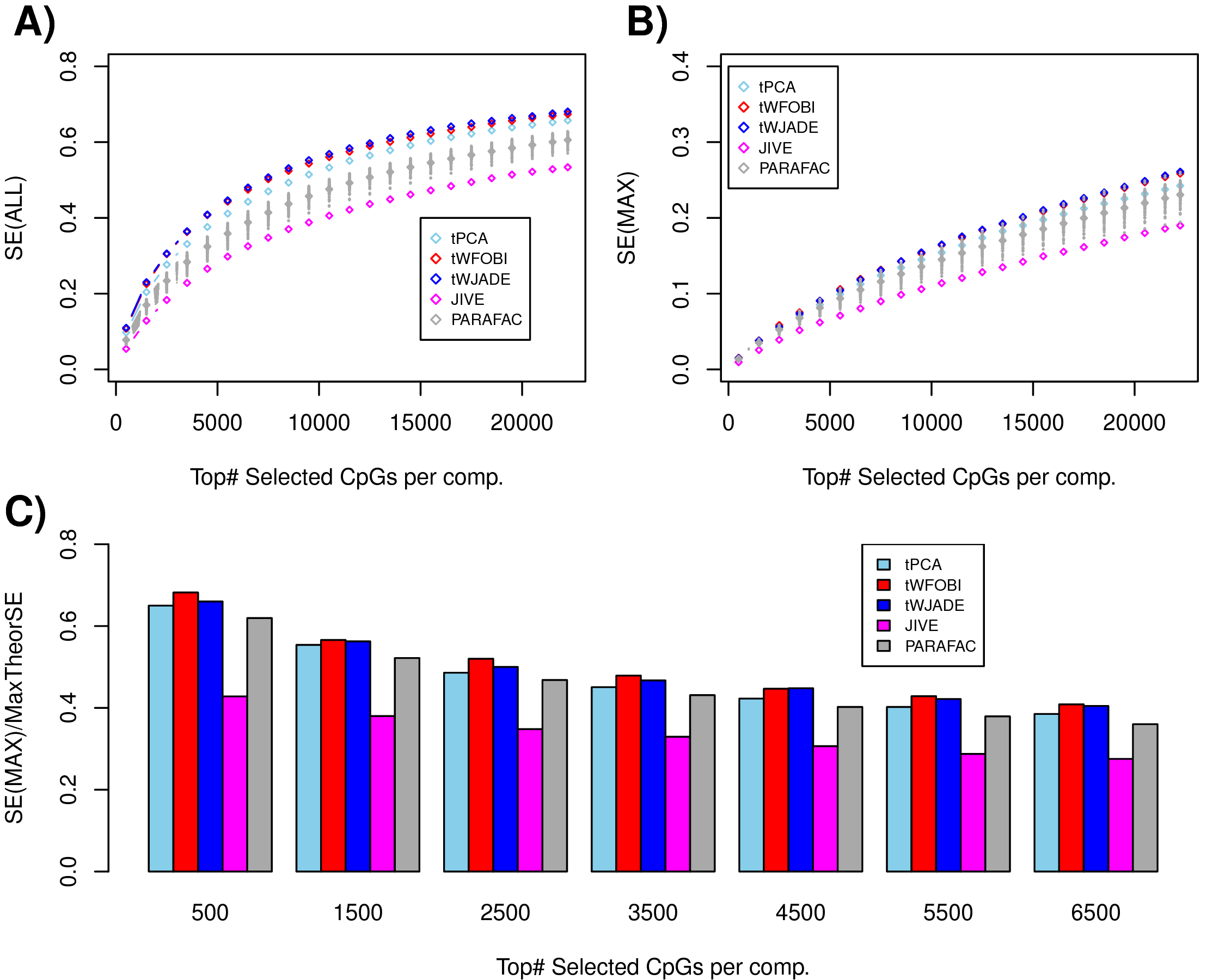


**SI fig.S6: tICA outperforms JIVE and PARAFAC in detecting mQTLs in the 3 blood cell subtype EWAS (as Fig.6 but with the results of 100 PARAFAC runs shown in panels A-B). A)** Plot of the overall sensitivity (SE(ALL),y-axis) against the number of top ranked CpGs selected in a component (x-axis) for 5 different multi-way algorithms, as applied to the 3 immune-effector cell-type EWAS. In the case of PARAFAC we show the results over 100 different runs. For all other algorithms results were stable between runs. **B)** As A) but now for the maximum sensitivity attained by any single component (SE(MAX),y-axis). **C)** Barplot of the maximum sensitivity attained by any single component expressed as a fraction of maximum possible value given the number of selected top-ranked CpGs per component (average shown for PARAFAC).


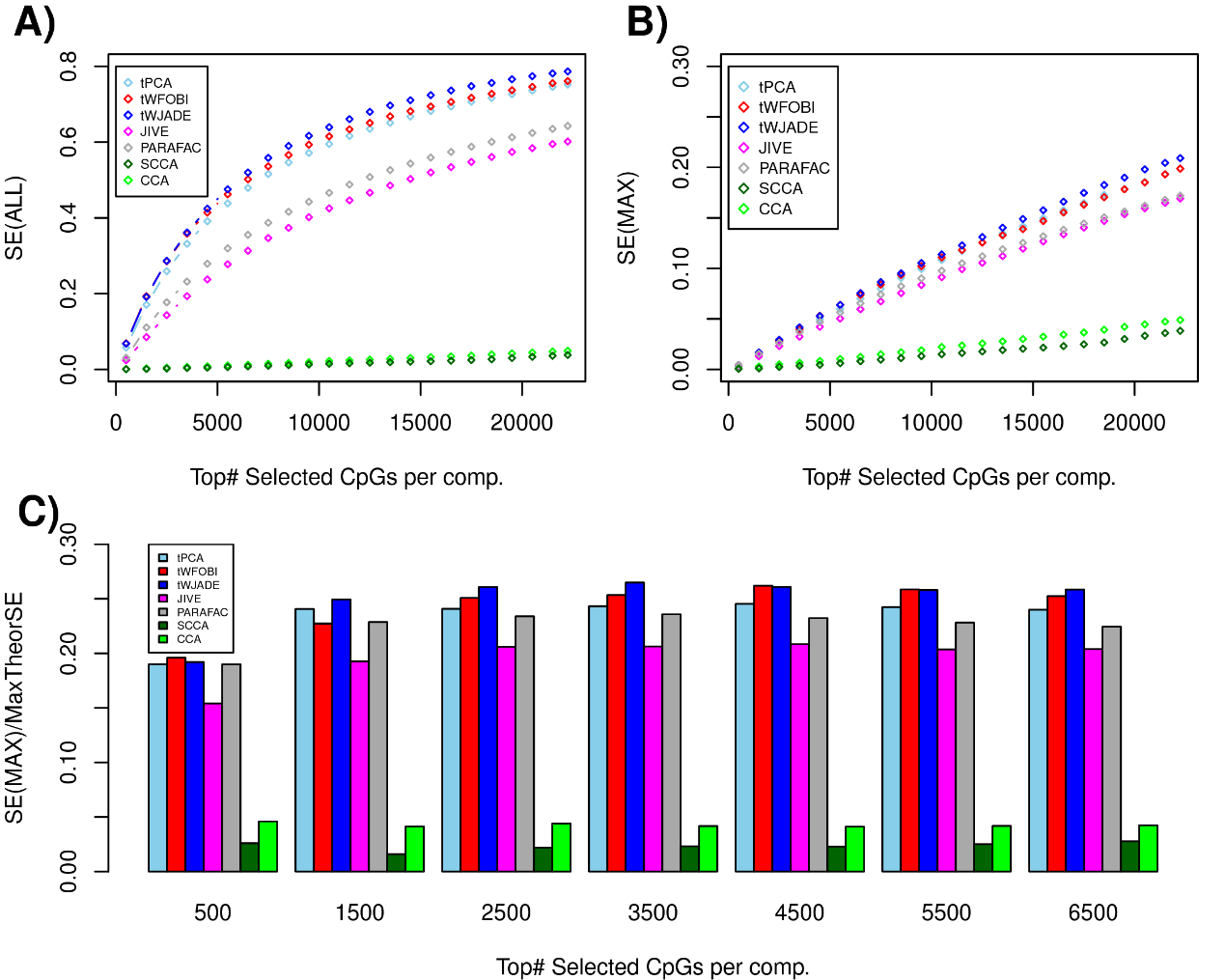


**SI fig.S7: Comparison of the sensitivity of multi-way algorithms to detect mQTLs in the buccal-blood EWAS. A)** Plot of the overall sensitivity (SE(ALL),y-axis) against the number of top ranked CpGs selected in a component (x-axis) for the 7 different multi-way algorithms, as indicated. **B)** As A) but now for the maximum sensitivity attained by any single component (SE(MAX),y-axis). **C)** Barplot of the maximum sensitivity attained by any single component expressed as a fraction of maximum possible value given the number of selected top-ranked CpGs per component. We note that for CCA and SCCA, only canonical vectors carrying significant variance were selected (as implemented in PMA R-package), which resulted in only one canonical vector being selected and hence the reduced performance.

**SUPPLEMENTARY TABLES**

| **IC (tWFOBI)** | **#Genes**  **(CopyN-Exp)** | **#Genes**  **(Meth-Exp)** | **Loci** | **P-value** |
| --- | --- | --- | --- | --- |
| IC-33 | 81 | 0 | 11q12-14,q21-24 7q22 | 1e-6 |
| IC-34 | 140 | 1 | 12q13-15,q23-24 | 3e-9 |
| IC-35 | 193 | 34 | 1q12,q21-25,q31-32,q41-42 | 2e-10 |
| IC-36 | 119 | 0 | 1p13,p21-22,p31-36 | 0.001 |

**table S1: Application of tWFOBI to the multi-omic colon TCGA data set.** Table summarizes results for 4 ICs which correlated with normal/cancer (N/C) status. Columns label the IC, the number of genes with CNV or DNAm driving mRNA expression (CopyN-Exp & Meth-Exp), the enriched cytoband loci and the P-value of association with cancer.
